# Internal audits as a tool to assess the compliance with biosecurity rules in a veterinary faculty

**DOI:** 10.3389/fvets.2023.960051

**Published:** 2023-03-02

**Authors:** Marie-France Humblet, Claude Saegerman

**Affiliations:** ^1^Unit Biosafety, Biosecurity and Environmental Licenses, Department for Occupational Protection and Hygiene, University of Liège, Liège, Belgium; ^2^Veterinary Science Epidemiology and Risk Analysis Research Unit (UREAR-ULiège), Fundamental and Applied Research for Animals and Health Center (FARAH), Department of Infectious and Parasitic Diseases, Faculty of Veterinary Medicine, University of Liège, Liège, Belgium

**Keywords:** standard operating procedure (SOP), prevention, awareness, zoonoses, compliance, quality control, biosecurity, hygiene

## Abstract

**Introduction:**

The present paper proposes a tool to follow up the compliance of staff and students with biosecurity rules, as enforced in a veterinary faculty, i.e., animal clinics, teaching laboratories, dissection rooms, and educational pig herd and farm.

**Methods:**

Starting from a generic list of items gathered into several categories (personal dress and equipment, animal-related items, infrastructures, waste management, management of material/equipment and behavior), a checklist was created for each sector/activity mentioned above, based on the rules and procedures compiled in the Faculty biosecurity standard operating procedures. Checklists were created as Excel™ files. For each sector, several sheets were elaborated, i.e., one per specific activity: for example, the following sheets were created for the equine clinic: class 1-2 hospitalization (class 1 = non-infectious conditions; class 2 = infectious disease with a low or non-existent risk of transmission), class 3 hospitalization (class 3 = infectious disease with a moderate risk of transmission; these patients are suspected of having an infectious disease and being contagious for other patients and/or for humans) and consultation.

**Results:**

Class 4 area, which corresponds to the isolation unit and aims at housing patients suffering from infectious diseases with a significant risk of transmission (including notifiable conditions), was not audited at that period, as it was undergoing renovation works. The audit relied on observations performed by a unique observer to ensure standardization. Observed items were presented as yes/no and multiple-choice questions. A scale from 0 to 3 or 4 (depending on the item) allowed scoring each item, i.e., 0 corresponding to 100% compliance with the procedure and the highest score to the worst situation. A median and average global score was also estimated by category and by activity.

**Discussion:**

The methodology described in the present paper allows estimating the compliance with biosecurity standard operating procedures in a specific sector and/or for a given activity. The identification of criteria needing improvement is a key point: it helps prioritizing actions to be implemented and awareness raising among people concerned. Regular internal auditing is an essential part of a biosecurity plan, the frequency being conditioned by the risk linked to a specific activity or area (i.e., more frequent audits in risky situations).

## 1. Introduction

The World Organization for Animal Health (OIE) defines biosecurity as “a set of management and physical measures designed to reduce the risk of introduction, establishment and spread of animal diseases, infections or infestations to, from and within an animal population” (1). It relies on the concept of the five B's, i.e., bio-exclusion, bio-compartmentation, bio-containment, bio-prevention and bio-preservation, as developed by Saegerman and collaborators (2). Prevention is thus a strong pillar in order to protect animal health, human health, especially when considering zoonoses, and the environment, through the One Health concept.

Biosecurity relies partly on the organization of infrastructures but also on the elaboration, implementation and respect of procedures. Veterinary practitioners and students are a population particularly at risk in terms of zoonoses (3, 4), due to their close contacts with sick animals.

To date, not much has been done or published regarding this topic (i.e., assessment of biosecurity in veterinary teaching hospitals). Escudero and collaborators (5) defined a biosecurity audit as the objective inspection and evaluation, through a complex transversal process, of current measures and highlights of weaknesses of the system. The audit is made of four main steps (5): (i) anterior data and information collection on the place to audit (allowing to assess *a priori* risks and map critical points, procedures or areas), (ii) biosecurity survey (mostly relying on checklists), (iii) on site visit and observations, and (iv), final diagnostic report and feedback to the audited company. An audit is thus an objective inspection and evaluation that checks biosecurity measures and identifies weaknesses. The report provides also recommendations in order to improve the situation and critical points or areas. To date, biosecurity audits are mainly implemented in livestock production systems such as pig and poultry industries. Indeed, in EU Member States, thus in Belgium as well, several biosecurity measures are now required through legislation, i.e. the “Animal Health Law” (6). Additionally, food authorities advocate or even require enterprises to set up a self-monitoring of their activities and procedures, as part of a quality control approach.

The present paper describes a methodology based on biosecurity checklists to assess compliance with biosecurity rules in a veterinary faculty (Liège University, Belgium). In that perspective, internal audits were implemented in different sectors of the Faculty of Veterinary Medicine where biological risks are assessed. Two case studies are presented to illustrate the concept, i.e., a specific area of the equine hospital, named “risk class 3”, and the Virology teaching biosafety level (BSL) 2 laboratory. These two case studies were selected as they illustrate two contrasted contexts: (i) a virology teaching lab, where biosecurity (in the context of contained use) is easy to comply with and where the staff is well present to guide the students step by step, and (ii), a context where students are most of the time left on their own in the clinic (the staff pays more attention to the patients rather than to students).

## 2. Materials and methods

As mentioned above, the method relies on the performance of an internal audit through the use of biosecurity-related checklists. The referential from which the checklists were elaborated was the Faculty Manual of Biosecurity Standard Operating Procedures (SOPs) (7). This manual was assessed and approved by the European Association of Establishments for Veterinary Education. The Biosecurity SOPs are structured by sector of activity (e.g., clinics—Equine, Ruminant, small animals—pig farm, experimental farm, food science, anatomy, teaching labs and diagnostic activities). Different sector-specific checklists and activity-specific checklists within a same sector were elaborated. They were structured according to the Faculty biosecurity website (8), thus divided into different categories of components or items:

- Persons, such as dress code and personal equipment
- People behavior, such as hand hygiene and movements within the facilities
- Animals, such as movements within the facilities
- Premises, such as cleaning and disinfection
- Waste management
- Material and equipment

Audited persons were students and staff members, i.e., scientific staff, technical staff and workers. The audit relied mainly on observations performed by a unique observer, to ensure standardization, but also on very short interviews of technical staff and workers. It did not consist only of one-time visits of infrastructures and observations, but also of several hours of observations of the way people behave and comply with required procedures. The duration of observations corresponded to the length of time spent by the students in the clinic or performing a specific activity (information specified in both case studies): observations started from the moment they entered the clinic or practical room until they left it, which represents a good overview of potentially at-risk behavior at all steps of the process.

Observed items were assessed as multiple choice or yes/no questions. Examples of checklists are presented in Supplementary material 1, 2. For multiple-choice questions, a score from 0 to 3 or 4 (depending on the item) was allocated to each item, i.e., 0 corresponding to 100% compliance with the procedure and the highest score for very low compliance. For example, in the Equine Clinic, the identification of the class of risk (see below–section 2.1–for definition) and disease diagnostic should be written on the box door; the scoring is thus the following: 0 = risk class plus disease diagnostic, 1 = risk class only, 2 = disease diagnostic only and 3 = no identification of risk nor disease diagnostic. For what binary questions are concerned, a score of 0 corresponded to the compliance with Biosecurity SOPs and a score of 3 to the non-compliance (in order to put them on a same footing as multiple-choice questions). No weighting process was performed, thus all items of the audit were allocated the same weight.

Data were encoded in an Excel™ datasheet. One sheet was elaborated per activity for a given sector (Supplementary material 1, 2). For example, in a particular clinic, different activities take place, such as consultation, hospitalization [regular vs. isolation facilities], surgery and outpatient activities. Median and mean scores are presented for each category of item, and per activity.

### 2.1. Case study 1–Class 3 of the Equine Clinic

In the Equine Clinic of Liège University Hospital, four classes of risk have been defined as a function of the pathogens/diseases involved (9):

- Class 1 = non-infectious conditions.
- Class 2 = infectious disease with a low or non-existent risk of transmission.
- Class 3 = infectious disease with a moderate risk of transmission; these patients are suspected of having an infectious disease and being contagious for other patients and/or for humans.
- Class 4 = infectious disease with a significant risk of transmission; any patient suspected of suffering from a notifiable disease falls in this category.

Class 3 patients are hospitalized in a dedicated aisle of the clinic and handled with barrier nursing precautions (9). Barrier nursing is a concept intending to protect other patients and/or the medical staff from contamination. It relies on the implementation of a “barrier” between the patient and medical staff in order to prevent cross-contamination of the body, clothing and footwear, which, in turn, decreases the risk of nosocomial transmission to other patients (7, 10). Barrier nursing precautions include, among others (7):

- Visible information on the animal health status (displayed on the box door)
- Wearing specially designated personal attire (e.g. specific/additional personal protective equipment [PPE] such as gloves, disposable overalls, cover-boots)
- Using material and equipment totally dedicated to the animal (e.g. for horses: halter, rope and examination equipment such as thermometer and stethoscope)
- Minimizing the movements of patients, ensuring the ‘onward march' and avoiding unnecessary contacts with them.
- Hospitalization in a separate unit (but not in isolation), if possible, with implementation of foot baths/mats.
- Management of waste as being biologically contaminated.
- Appropriate decontamination protocols when the patient leaves the unit.

Here are some examples of conditions classified as class 3: fever and/or leukopenia of unknown origin, viral respiratory disease (cough, nasal discharge [<2 weeks], possibly with fever), infections with *Rhodococcus equi* (foals <10 months with respiratory problems and fever), diarrhea without fever and/or leukopenia, non-surgical digestive problem with hemorrhagic reflux or non-hemorrhagic reflux with fever and/or leukopenia, methicillin-resistant *Staphylococcus aureus* (MRSA) or other multi-resistant bacterial infections, and contagious skin infections (dermatophytosis, dermatophilosis, chorioptic mange, phtiriasis and other parasitic conditions) (9).

The premises consist in a unit of four boxes and a second unit of two boxes. An automated high-speed shutter separates the class 3 from the rest of the clinic.

People audited were mainly clinicians (interns, residents and senior clinicians), technicians, stable staff, and students in 2nd and 3rd year of a master in veterinary medicine program (GVM2 and GVM3).

The checklists elaborated for that specific sector are available as Supplementary material 1.

### 2.2. Case-study 2–Practical virology laboratory sessions

The practical virology sessions consist in 2 periods of 2 h each: students in 2nd year of the bachelor in veterinary medicine (BVM2) handle samples in the biosafety cabinet during one of the 2 periods. After a briefing on the organization of each practical session (in the lab—clean zone), students are split into several groups of 2 to 3 students. A first group works using the biosafety cabinet (BSL2 activity) while the other group remains in the lab's “clean zone” for the observation of fixed microscope slides, a BSL1 activity. Two staff members supervise the activities, i.e., a scientist and a technician.

Checklists for each category of items are available in Supplementary material 2.

### 2.3. Data analysis

As samples were small (pilot study), a basic analysis was performed in Excel™ software through the estimation of mean and median scores. Both are needed to better describe the data.

Because the mean uses all observations, it will be affected by extreme values (outliers) in a data set. The mean is thus not the measure of choice on its own for data that are severely skewed or have extreme values in one direction or another (11). Indeed, as a result of one extremely large value, the mean could be larger than all values in the distribution except the extreme value (the “outlier”) (11). Nevertheless, in the present context, such characteristic considered as negative could directly draw attention on a special category of items, if it reaches a maximal score (corresponding to a total non-compliance); that category of items should thus need to be investigated more deeply.

The advantage of medians is that it is a good descriptive measure, particularly for data that are skewed, because it is the central point of the distribution and it is not generally affected by one or more outliers (11); however, in the present context, an item for which compliance is totally lacking, might go unseen if considering only medians, as it tends to ignore extreme values.

## 3. Results

In both case studies, results are presented as average and median scores, as the aim of the audit was to assess the compliance with required biosecurity rules and to identify components to be improved. No intra- nor inter-category weighting process was applied. The results of observations for both activities are presented below.

### 3.1. Case study 1–Class 3 of the Equine Clinic

The audit of the Equine Clinic-class 3 was conducted on December 5, 2017, over a half-day period, after a suspicion of nosocomial MRSA problem. The duration of observations corresponded to the length of time students spent in the Clinic. It targeted the Equine Clinic-class 3 as MRSA patients are hospitalized in that area, according to the Clinic classification of risks in as detailed in the Material and Methods section. The audited persons were: 10 students, 10 clinicians, 1 technician and 3 members of the stable staff.

**Figure 1:**
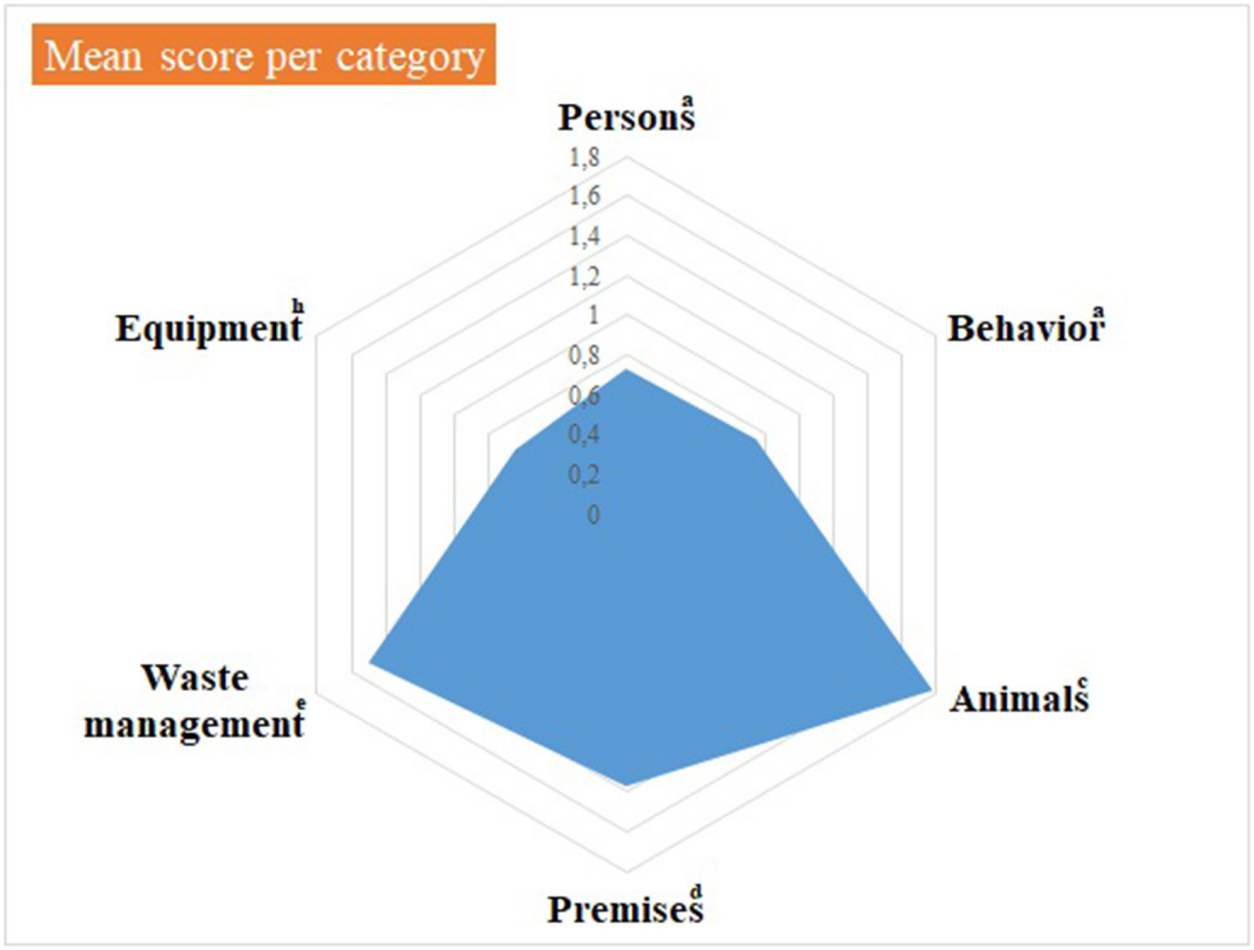
Illustration of the mean score, per category of items, of the internal biosecurity audit performed in the Class 3 of the Equine Clinic. ^a^For the categories “Persons” and “Behavior”, the number of observations (= the number of people observed per category, i.e. students, scientific staff members and stable staff members) was 7–10 students of 2nd and 3rd master in veterinary medicine (GVM2 and GVM3), 10–12 scientific staff members and 3 stable staff members. ^c^For the category “Animals”, the number of observations was: animals/boxes = 1, consultation = 1, feeding habits = 1 and animal movements = 7. ^d^For the category “Premises”, the number of observations was: boxes = 7, cleaning order = 1, hand hygiene = 5, boot cleaning =1, foot baths/mats = 5, doors = 1–5, cleaning equipment =1 and procedure of cleaning and disinfection = 1. ^e^For the “Waste management” category, the number of observations (= number of waste equipment present in the facilities) was 9. ^h^For the “Equipment” category, there was one observation, i.e. the procedure in force for cleaning and disinfecting. The number of observations per item appears in Supplementary material 3.

**Figure 2:**
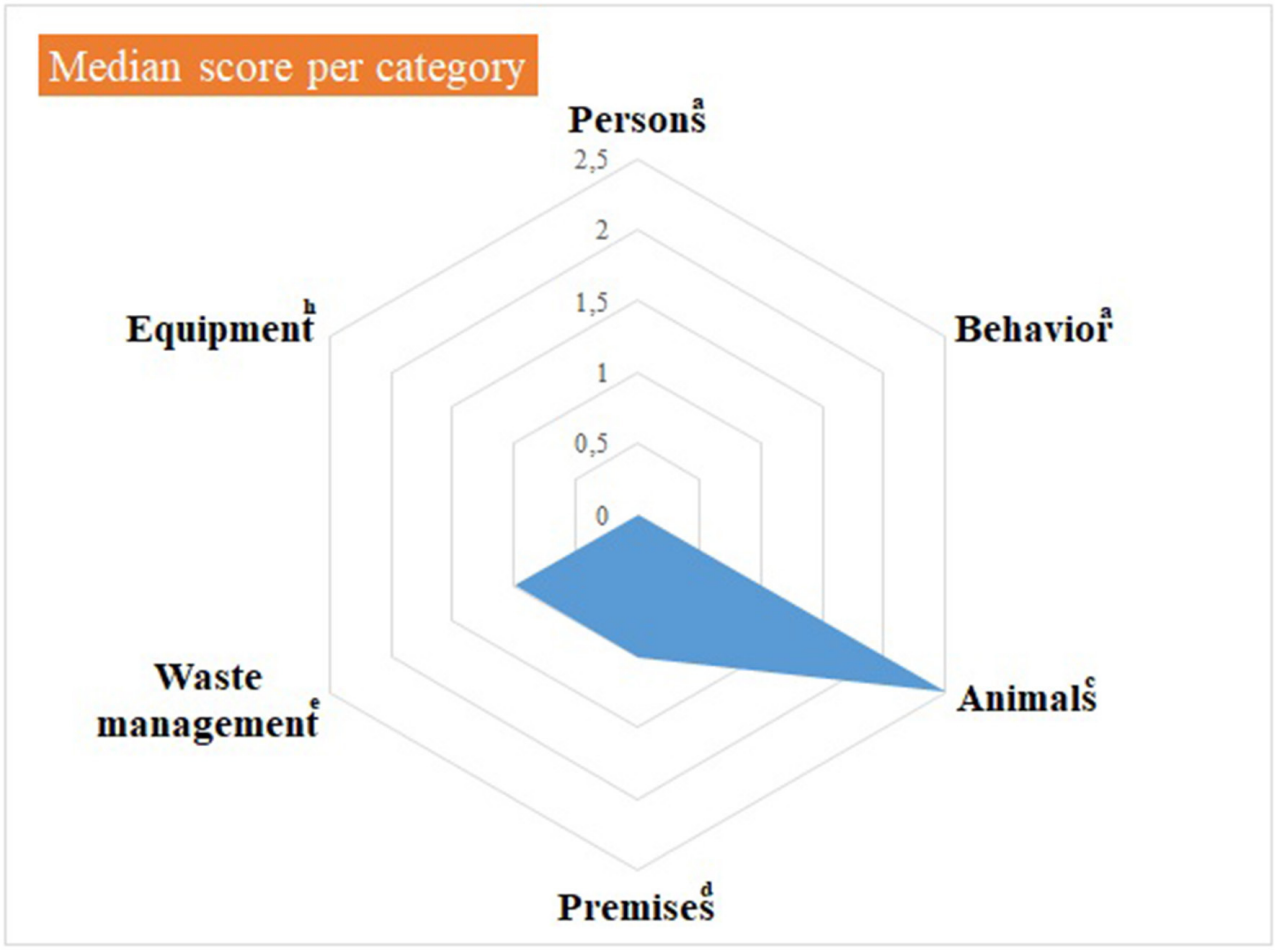
Illustration of the median score, per category of items, of the internal biosecurity audit performed in the Class 3 of the Equine Clinic. ^a^For the categories “Persons” and “Behavior”, the number of observations (= the number of people observed per category, i.e. students, scientific staff members and stable staff members) was 7–10 students of 2nd and 3rd master in veterinary medicine (GVM2 and GVM3), 10-12 scientific staff members and 3 stable staff members. ^c^For the category “Animals”, the number of observations was: animals/boxes = 1, consultation = 1, feeding habits = 1 and animal movements = 7. ^d^For the category “Premises”, the number of observations was: boxes = 7, cleaning order = 1, hand hygiene = 5, boot cleaning = 1, foot baths/mats = 5, doors = 1–5, cleaning equipment =1 and procedure of cleaning and disinfection = 1. ^e^For the “Waste management” category, the number of observations (= number of waste equipment present in the facilities) was 9. ^h^For the “Equipment” category, there was one observation, i.e. the procedure in force for cleaning and disinfecting. The number of observations per item appears in Supplementary material 3.

Median and mean scores, per category of items, are detailed in Supplementary material 3 and shown in Figures 1, 2, as well as in Table 1. Figure 1, Table 1 highlight some deficiencies in the category “animals”. They mostly concern the animals themselves, the consultation organization, feeding and animal movements. However, only one observation was performed for animal movements. All deficiencies observed are detailed in Tables 2A–F, per category.

**Table 1:** Class 3 of the Equine Clinic—mean and median scores per category of items.

**Category of items**	**Observations**		**Median score**	**Mean score**
	**Items observed**	***N*** (range)^*^		
Persons	GVM2/GVM3 students	7–10	0.00	0.73
	Scientific staff members	10–12		
	Stable staff members	3		
Behavior	GVM2/GVM3 students	3–4	0.00	0.75
	Scientific staff members	1–3		
	Stable staff members	1–2		
Animals	Animals	7	2.50	1.78
	Consultation	1		
	Feeding	1		
	Animal movements	7		
Premises	Boxes	7	1.00	1.37
	Cleaning order	1		
	Hand hygiene	5		
	Shoe/boot cleaning	1		
	Foot baths/mats	5		
	Unit door closed	5		
	Building door closed	1		
	Cleaning equipment	1		
	Cleaning and disinfection	1		
Waste management		9	1.00	1.50
Equipment		1	0.00	0.64

^*^N observations: for the categories “persons” and “behavior”, it correspond to the number of people who were observed (per category, i.e. students, scientific staff members and stable staff members). For the category “animals”, it corresponds either to the number of animals or boxes observed, to the number of consultations observed, to sub-items categorizing feeding habits and to the number of animal movements. For the category “premises”, it corresponds to the either the number of devices present in the facilities (for shoe/hand hygiene and cleaning). For the “waste management” category, it corresponds to the number of waste equipment present in the facilities. For the “equipment” category, it corresponds to the procedure in force for cleaning and disinfecting. The number of observations per item appears in Supplementary material 3. GVM, Master in Veterinary Medicine.

**Table 2A:** Biosecurity deficiencies observed when performing the internal audit in the Equine class 3 area—category “PERSONS”.

**Category of items**	**Score**				**Non-compliances observed**	**Risk**	**Recommendations**
**PERSONS**	**0**	**1**	**2**	**3**			
Dress and equipment	12	5	2	1	Students' overall clean but not buttoned up; staff not wearing any PPE^*^	Contamination of “city clothes” worn under the overalls	Wear PPE correctly
	12	8	0	0	Footwear: not appropriate (no rubber boots or safety shoes)	Avoiding walking through the foot baths/mats if inappropriate footwear	Wear clean rubber boots or safety shoes (easy to clean and disinfect)
	10	0	0	1	Long-sleeves disposable lab coat not worn systematically	Risk of contaminating other patients if underneath non-class 3 PPE gets contaminated	Wear the additional PPE, i.e., long-sleeves disposable lab coat
Entry of persons	17	1	1	2	Walking through the foot bath/mat set up near the box door is not systematic	Risk of spreading pathogens through footwear	Walking through any footbath/ mat is mandatory
	4	2	0	15	No hand hygiene upon entering the class 3 units	Risk of transfer of pathogens through hand contact	Hand hygiene is mandatory before entering the unit
	15	0	0	6	Long-sleeves disposable lab coat not worn	Contamination of PPE worn in the rest of the clinic ≥ risk of contaminating non-class 3 patients	Apply barrier nursing through wearing the additional PPE, i.e. long-sleeves disposable lab coat
Between two patients	11	0	0	5	No systematic glove changing	Risk of contaminating other patients	Change gloves between 2 patients
	11	0	0	5	No use of long sleeve disposable apron	Risk of contaminating other patients if underneath non-class 3 PPE gets contaminated	Wear the additional PPE, i.e. long-sleeves disposable lab coat
	5	6	0	5	No hand hygiene	Risk of hand contamination and further spread of pathogens to other patients or through fomites	Hand hygiene is mandatory between two patients and upon leaving the area
	7	2	0	7	Inappropriate frequency of hand hygiene
Exit of persons	3	0	0	3	No hand hygiene
	11	0	0	5	No use of disposable gloves		Wear PPE (i.e., gloves) for any contact with a class 3 patient.
	8	0	0	8	Fate of long sleeve disposable apron: no use	Risk of contaminating other patients if basic PPE is not protected and gets contaminated	Wear the additional PPE, i.e., long-sleeves disposable lab coat
	5	7	0	4	No systematic hand hygiene upon leaving the area	Risk of hand contamination and further spread of pathogens	Hand hygiene is mandatory upon leaving the class 3 area

^*^PPE, Personal protective equipment; N people observed, 7–10 students, 10–12 scientific staff members and 3 stable staff members; GVM, Master in Veterinary Medicine.

**Table 2B:** Biosecurity deficiencies observed when performing the internal audit in the Equine class 3 area—category “BEHAVIOR”.

**Category of items**	**N observations per score**				**Non-compliances observed**	**Risk**	**Recommendations**
**BEHAVIOR**	**0**	**1**	**2**	**3**			
General	8	0	1	0	Presence a dog roaming free in the yard	Risk of the dog entering the class 3 unit and plays the role of vector of pathogens (spread)	No dogs are allowed on site
Food and beverage consumption	7	0	2	0	Consumption of beverages in the clinic	Risk of a person to get contaminated by a zoonotic pathogen	The consumption of food and beverages should be done in the cafeteria (not in the clinic)
Hand hygiene	2	2	1	2	No-compliance with the standard hand hygiene method (use of clear water only or no hand hygiene at all)	Risk of hand contamination with pathogens that might be zoonotic and risk of cross-contamination of other patients through contact with contaminated hands.	Apply the recommended hand washing method or sanitizing when appropriate (and as often as possible)
	4	0	0	2	No systematic hand hygiene after wound care or bandage change
	3	1	0	0	Hand hygiene after catheter placement
	3	1	0	0	Hand hygiene after eye care
	1	2	1	0	Hand hygiene after each patient
			1		No hand hygiene after cleaning/disinfecting the boxes
	1	1	0	3	Hand hygiene upon exiting the clinic

For that category, N people followed, 3 students, 3 scientific staff members and 1 stable staff member; GVM, Master in Veterinary Medicine.

**Table 2C:** Biosecurity deficiencies observed when performing the internal audit in the Equine class 3 area—category “ANIMALS”.

**Category of items**	**N observations per score**				**Non-compliances observed**	**Risk**	**Recommendations**
**ANIMALS**	**0**	**1**	**2**	**3**			
Animals	0	5	0	2	Identification on the box door (class of risk and disease diagnostic): no identification	Lack of appropriate protective measures (or caution), especially if zoonotic pathogen	Identification of risk class and disease diagnostic make aware on the importance of preventive measures
	2	3	0	0	Medicine left in front of the box only (no identification)	Risk of contaminating other patients if medicine is shared	Medicine should be left in a box fixed on the box door
	0	0	0	7	No identification of the risk class on the patient	No information on the infectious risk and necessity of specific measures (hygiene, potential zoonotic risk)	Identification of the risk class on the patient (e.g., colored halter) or on its file
Feeding				1	Minimal amounts of fodder in the area but open access	Risk of pest contaminating fodder stored in the area	Prevent access of pests to feeding sources
				1	Straw, fodder and cereals stored in the barn, but open doors		
			1		Control of rodents in the hospital only	Risk of presence of rodents, and associated risks in the barn	Implement rodent control in the hospital and in the barn
Movements	0	0	1	0	Occasional movements of a class 3 patient	Risk of spreading the pathogen outside the box	Movements of class 3 patients are restricted (only for exceptional reasons such as complementary examination in the Imaging Unit)
				1	No prevention of pathogen spread outside the box: (a) no cleaning of horse feet before leaving the box, (b) no scrubbing of horse feet with 0.5% chlorhexidin, (c) no overshoes worn, (d and e) no cleaning nor disinfection of the horse's path if necessary		Avoid pathogen spread by: (a) cleaning and disinfection of horse feet before leaving the box (b) put overshoes on horse feet before entering the Imaging Unit (c and d) cleaning and disinfection of the horse's path if necessary

N, number of; the number of observations varies according to the item. N, 7 for animals; 1 for items related with feeding and 1 animal movement.

**Table 2D:** Biosecurity deficiencies observed when performing the internal audit in the Equine class 3 area—category “PREMISES”.

**Category of items**	**score**				**Non-compliances observed**	**Risk**	**Recommendations**
**PREMISES**	**0**	**1**	**2**	**3**			
General information	0	2	0	2	Identification = infectious diagnosis only (no risk class)	No knowledge of the class of risk (and associate preventive measures to be implemented)	Identification through class of risk and diagnosis
	0	2	2	3	Color lines not visible	Risk of not complying with biosecurity rules in force in the class 3 area	Increase visibility of color lines
				1	Door knobs never disinfected	Risk of hand contamination	Implement at least daily disinfection of door knobs
For hand hygiene	3	0	2	0	Antibacterial soap not always available (dispensers empty)	Risk of hand contamination	Availability of antibacterial soap should be guaranteed
	4	0	1	0	Paper for hand drying not always available	No correct drying before disinfection	Availability of paper should be guaranteed
Footwear cleaning				1	No boot-washer	Risk of transporting pathogens on footwear if not cleaned (and disinfected)	Equip the premises with at least one boot-washer to ensure correct cleaning wearing boots vs. booties is strongly advised as well, as easier to clean
Foot baths/mats	3	0	1	1	Foot baths/mats not present in every unit	Be sure a foot bath/mat is present at the entrance to each unit
	4	0	1	0	Footbath/foot mat macroscopically dirty	Organic matter inactivates many disinfectants ≥ footwear not correctly disinfected	Need to be free of organic matter in order for the disinfectant to be efficient
Door closing	4	1	0	0	Unit doors not always closed systematically	Risk of pests entry (with further spread of pathogens)	Unit doors should be closed at any time.
			1	Building door remains open all the time	Risk for pests, dogs, cats, etc. to enter the area, although the access is restricted	Building door should be closed at any time
Cleaning and disinfection (boxes, consultation room, corridors, cleaning equipment, B41 courtyard, indoor horizontal surfaces)	Lack of compliance with the standard method of cleaning - No detergent or detergent used after disinfection (boxes) - Garden hose only (wheelbarrow, cleaning tools) - Dry cleaning only (sweeping) (corridors) - No cleaning before disinfection (boxes) - Cleaning frequency too low (consultation room, corridors, covered walkaway, building courtyard)					Risk of non-effectiveness of the decontamination process: organic matter can inactivate many disinfectants, risk of pathogen persistence in the environment and further spread through fomite	Follow the standard procedure for cleaning and disinfection (see Supplementary material 1): cleaning before disinfection, use the recommended biocide, follow the manufacturer's instructions with regards to the contact time and frequency of cleaning and disinfection
	Lack of compliance with the standard method of disinfection: - Use of a disinfectant other than the one recommended, (but registered for use and efficient) - Standard contact time of 20 min (for all disinfectants used) - Frequency of disinfection too low (consultation room, corridors, covered walkaway, building courtyard, and indoor horizontal surfaces)					The disinfection process might not be guaranteed and efficient = > risk of pathogen persistence in the environment and further spread
	No additional PPE to perform cleaning and disinfection					Chemical risk, in addition to the biological risk	Wearing an additional and appropriate PPE is recommended while performing the cleaning and disinfection process.

PPE, Personal protective equipment. For the item “cleaning and disinfection”, only one observation is included (the score of each item ranging from 1 to 3). A score of 3 was registered, for example, for the items “wearing additional PPE for cleaning and disinfection”, “frequency of cleaning” for cleaning tools and building courtyard, and “frequency of disinfection” for corridors, covered walkaway and indoor horizontal surfaces.

**Table 2E:** Biosecurity deficiencies observed when performing the internal audit in the Equine class 3 area—category “WASTE”.

**Category of items**	**Score**				**Non-compliances observed**	**Risk**	**Recommendations**
**WASTE**	**0**	**1**	**2**	**3**			
B2 waste containers	0	0	0	9	Absence of poster(s) reminding B2 waste management	Wrong waste management	Important to remind the correct management of biologically contaminated waste
	5	0	0	4	Exterior of B2 yellow containers sometimes dirty	Risk for waste collector (hand contamination)	Importance to keep the exterior of B2 waste containers as clean as possible (disinfection spraying before leaving the area is advised)
B2 waste containers for sharps	0	0	0	9	Small containers for sharps never used (sharps disposed of in large containers)	Risk for people handling large containers, especially if one tries to compact the contents with hands	Sharps must be disposed of in dedicated containers.
	0	0	0	9	Absence of poster(s) reminding the management of sharps	Risk for people who try to compact the waste container contents with hands, if not aware of the way to dispose of sharps.	Important to remind the correct management of sharps in order to avoid needle stick injuries.
	0	0	0	9	Absence of containers for sharp disposal	Containers for sharps must be present in each unit
Containers for B1 non-contaminated medical waste	2	2	2	3	Containers for B1 waste not used systematically when they should be	Risk of disposal of contaminated waste in these containers	Containers for B1 waste should be used systematically when appropriate
	6	0	0	3	Absence of containers for B1 waste	Risk of disposal in other containers	One containers for B1 waste should be present in each unit
General use of waste containers	3	0	0	6	In general, waste is not disposed of correctly and in appropriate containers	Risk of contamination and needle stick injuries	Waste management should be improved

B1 containers are used for disposal of non-contaminated medical waste and B2 containers for the disposal of biologically contaminated medical waste. Nine observations were considered for all items.

**Table 2F:** Biosecurity deficiencies observed when performing the internal audit in the Equine class 3 area—category “EQUIPMENT”.

**Category of items**	**Score**				**Non-compliances observed**	**Risk**	**Recommendations**
**EQUIPMENT**	**0**	**1**	**2**	**3**			
Equipment for animals			1		Animal's proper equipment often used in the class 3 area	Risk of contaminating the patient's equipment with nosocomial pathogens	Patient's own equipment should not be used in the class 3 area (left to the owner upon admission)
			1		The Clinic only provides occasionally blanket and halter	Risk of contaminating the patient's equipment if used in the class 3 area	Blankets and halters should be provided systematically upon patient's admission
Examination equipment		1			Standard disinfectant(s) used for examination equipment other than the one recommended but adapted	The disinfection process might not be 100% efficient and guaranteed	Use of standard disinfectant(s) recommended.
	1			Stethoscope only cleaned upon patient discharge	Risk of pathogen transfer if misused.	In the class 3 area, stethoscopes should be cleaned and disinfected after each use
	1			Stethoscope only disinfected upon patient discharge
			1	Equipment (cans, etc.) is never disinfected before leaving the area as it is supposed to remain in the class 3 area	Risk of pathogen transfer between the class 3 area and the rest of the clinic if equipment inadvertently taken out of the area	Even if dedicated to the class 3 area, equipment should be cleaned and disinfected systematically after use.
			1	No special treatment after a parasitic or a mycotic disease	Risk of environmental persistence of parasites and/or mycotic agents	A special treatment should be applied after a parasitic or a mycotic disease
Biological samples			1		Risk not specified on the sample carrying bag	Lack of appropriate preventive measures when handling the samples (risk of human contamination if zoonotic pathogen)	Identification and risk (i.e. class 3) should be clearly mentioned on the sample carrying bag

Scores concern one observation for each item of the category.

For the category “persons” (Table 2A), the main deficiencies were related to the PPE, i.e., students' overall not correctly worn, not always appropriate footwear, additional PPE (i.e., long-sleeves disposable lab coat) not worn systematically and gloves not changed systematically between two patients.

For the category “behavior” (Table 2B), apart from the presence of a dog roaming freely in the yard, the main deficiencies were related to the consumption of food and beverage in the clinic, and inappropriate hand hygiene, i.e., neither systematic nor following systematically the standard procedure. Regarding all items related to movements of animals, i.e. picking horse's feet before getting out of the box, scrubbing horse feet with 0.5% chlorhexidine before getting out of the box, putting overshoes on horse feet to go to the Imaging Unit, cleaning of the horse's path, and its disinfection if necessary, all of them got a maximum score of 3, but only one animal movement was observed.

The scores as presented in Table 2C concern the category “Animals”, identified as the one with major deficiencies (Table 1 and Figure 1). No identification of the infectious diagnosis was specified on the box door nor on the patient (wearing a colored halter) or its file. Access to feed and straw was continuously available, so pests could potentially use the area for shelter and food, and could act as pathogen vectors to other areas of the hospital. In addition, rodent control was only implemented in the hospital itself.

For the category “premises” (Table 2D), observations highlighted several deficiencies marked through a score different from 0). The risk class was not identified on doors and color lines were not visible, so restricted areas were not well identified, which is especially important for students. Doorknobs were never disinfected and no dispositive for washing footwear was present. Foot baths/mats were not observed everywhere expected or were dirty. The building door remained opened all the time, so pest, arthropod vectors (and dogs) could go in and out without any restriction and further spread pathogens in the rest of the clinic or in the vicinity. The cleaning and disinfection process were deficient (as specified in Supplementary material 1). Some items scored poorly (see Supplementary material 3), such as frequency of cleaning tools and building courtyard, standard disinfectant use for tools and covered walkaway, frequency of disinfection for corridors, covered walkaway and indoor horizontal surfaces. Furthermore, staff did not wear any adequate PPE while performing cleaning and disinfection.

The main deficiencies for waste management were (Table 2E): no reminding poster on the management of biologically contaminated waste, i.e., B2 waste, external side of B2 containers sometimes dirty (risk of contamination for future handlers), incorrect management of sharps (risk of needle stick injuries) and globally inappropriate use of waste containers.

Table 2F summarizes the deficiencies observed for the category “equipment”. The patient's own equipment was sometimes used, because the clinic did not provide it systematically (the material brought with the patient upon admission should be taken home by the owner, and neither left nor used for the patient while it is hospitalized in the clinic; all equipment should be provided by the Clinic). No specific environmental treatment was applied after housing a patient with parasitic or mycotic disease (risk of contaminating the next patient housed in the box). The risk class of the patient was not specified on sample transportation bags, so the lab was not directly informed about the risk and necessary biosafety measures. Equipment was not disinfected before leaving the class 3 area to be brought to the main hospital for a deeper decontamination (the risk of pathogen exiting *via* equipment was considered limited), stethoscopes were not cleaned and disinfected after each use (acceptable risk as they are patient-specific). The disinfectant used for examination equipment was not the one recommended in the SOPs, but was adapted to a given risk.

The internal audit and observations of practices highlighted some possibilities for improvement for each category. After the audit, for a given sector, a feedback was provided through a report including several recommendations (see Tables 2A–F).

### 3.2. Case-study 2–Practical virology sessions

The internal audit of the two practical virology sessions were performed on December 9 and 11, 2017; each session lasted 2 h. Two staff members (one scientist and one technician) and 14 BVM2 students were audited, during both sessions. Students were split into two subgroups and each subgroup performed a BSL2 activity during one session (six students on the first session and eight during the second session, as one student was missing during the first session).

**Figure 3:**
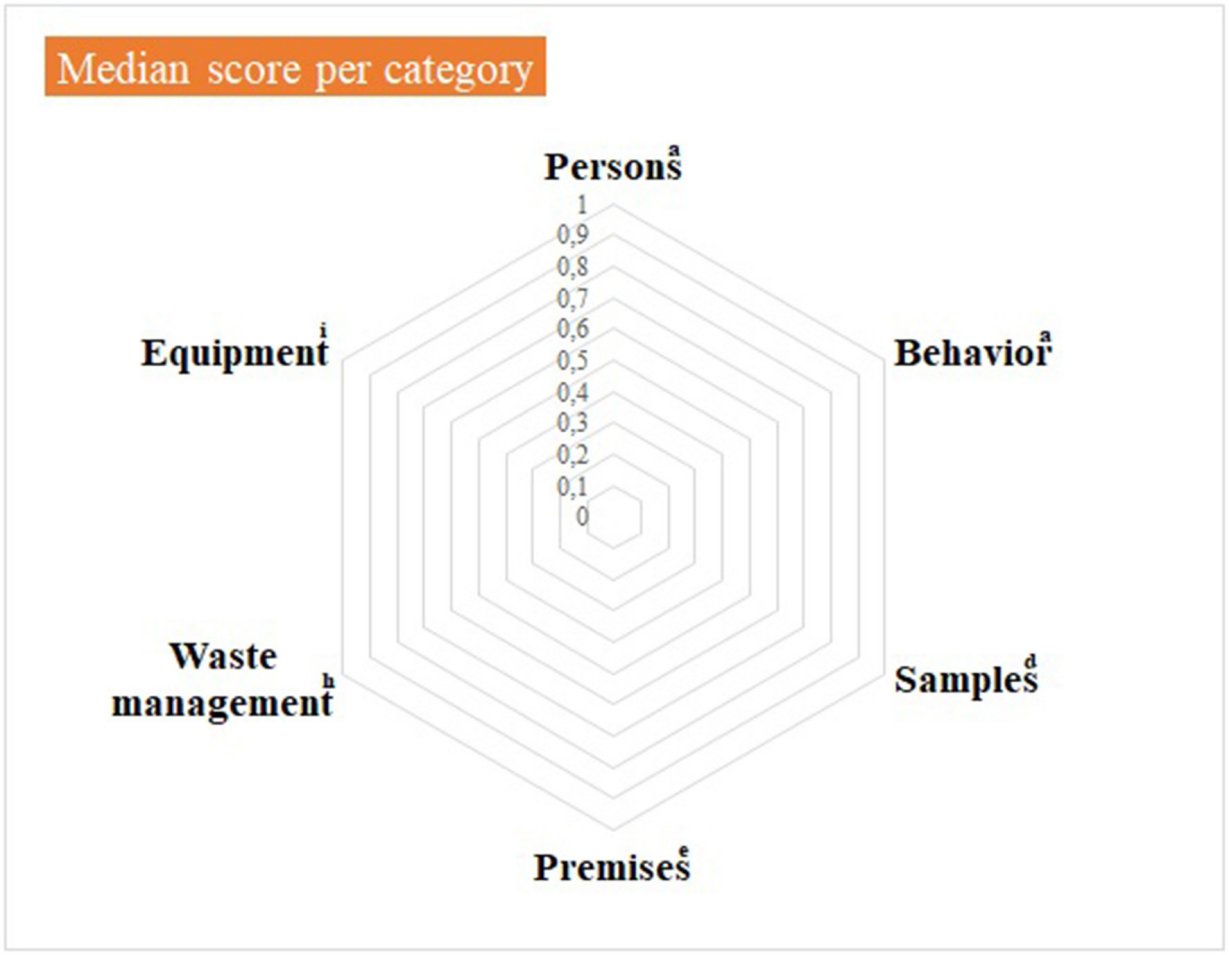
Virology practical sessions—median score per component of the internal biosecurity audit. ^a^For the categories “Persons” and “Behavior”, N observations (= number of people observed per category) = 14 students of 2nd bachelor in veterinary medicine (BVM2) and 4 scientific and technical staff members. ^d^For the category “Samples”, N observations = 1; ^e^For the category “Premises”, N observations = 1 (the lab itself, its preparation for practical activities and cleaning and disinfection process): ^h^For “Waste management”, N observations = 1 (waste disposal equipment present in the lab and anteroom). ^i^For “Equipment”: N observations = 1 for the management of the lab equipment (lab equipment and preparation procedure) and N = 14 for movements of equipment. The number of observations per item appears in Supplementary material 4.

**Figure 4:**
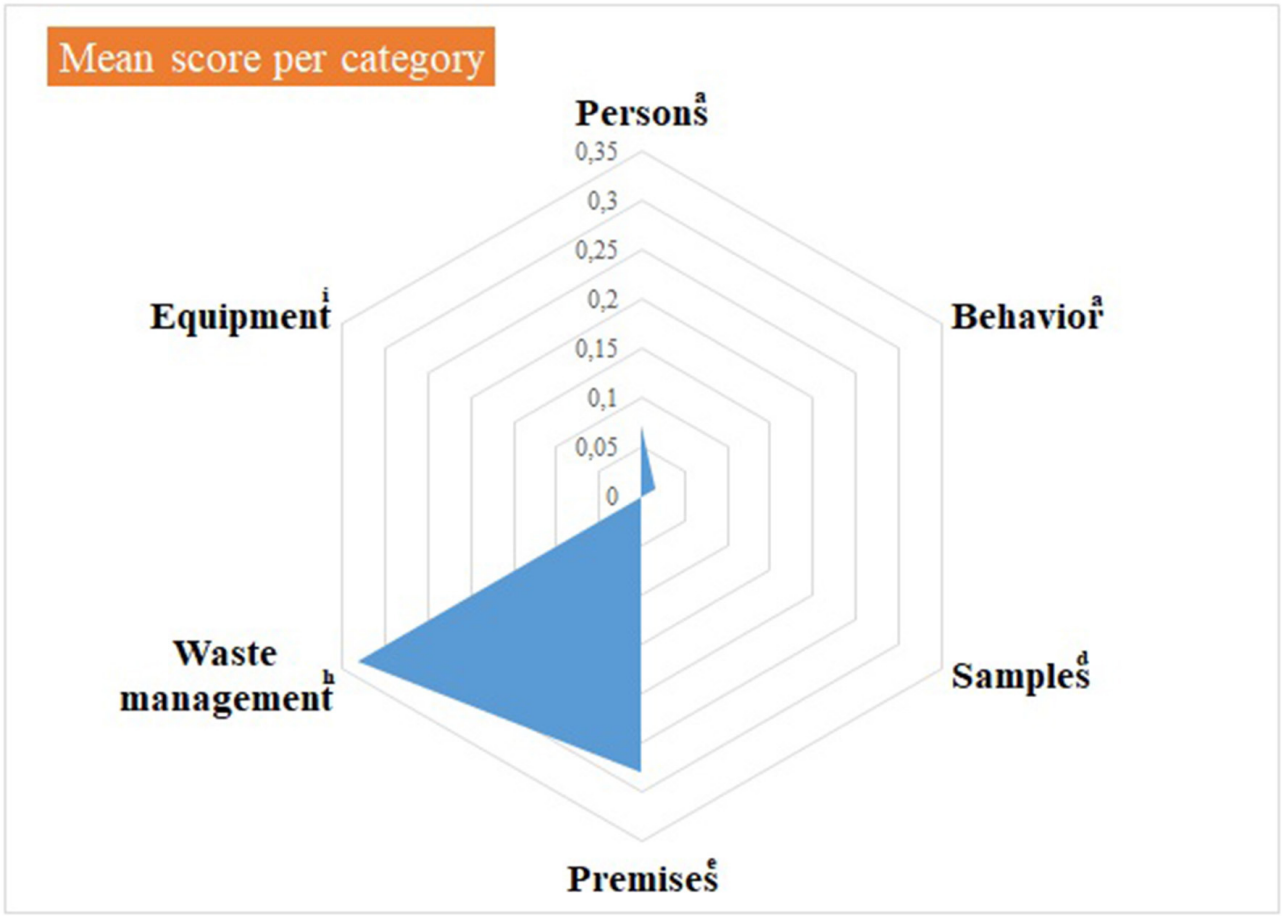
Virology practical sessions—mean score per component of the internal biosecurity audit. ^a^For the categories “Persons” and “Behavior”, N observations (= number of people observed per category) = 14 students of 2nd bachelor in veterinary medicine (BVM2) and 4 scientific and technical staff members. ^d^For the category “Samples”, N observations = 1; ^e^For the category “Premises”, N observations = 1 (the lab itself, its preparation for practical activities and cleaning and disinfection process): ^h^For “Waste management”, N observations = 1 (waste disposal equipment present in the lab and anteroom). ^i^For “Equipment”: N observations =1 for the management of the lab equipment (lab equipment and preparation procedure) and *N* = 14 for movements of equipment. The number of observations per item appears in Supplementary material 4.

Median and mean scores, per category, are detailed in Supplementary material 4 and illustrated in Figures 3, 4, as well as in Table 3.

**Table 3:** Practical virology sessions—mean and median scores per category of items.

**Category of items**	**Observations**		**Median score**	**Mean score**
	**Items observed**	***N*** (range)^*^		
Persons	BVM2 students Scientific and technical staff	14 4	0.00	0.07
Behavior	BVM2 students Scientific and technical staff	14 4	0.00	0.0l2
Samples		1	0.00	0.00
Premises		1	0.00	0.28
Waste management		1	0.00	0.33
Equipment	Lab equipment Preparation procedure Movements of equipment	1 1 14	0.00	0.00

BVM, Bachelor in Veterinary Medicine; ^*^for the categories “persons” and “behavior”, the number of observations corresponds to the number of people observed, per category. For the category “samples”, it corresponds to their identification, storage and movements, but also all that concerns accidental spills. For the category “premises”, it corresponds to the lab itself, its preparation for practical activities and the cleaning and disinfection process. For the “waste management” category, it corresponds to the waste disposal equipment present in the lab and anteroom. For the “equipment” category, it corresponds to the management of the lab equipment and its eventual movements. The number of observations per item appears in Supplementary material 4.

Median null scores correspond to a complete compliance with Biosecurity SOPs, as illustrated in Figure 3. Average scores were better if compared with the Equine class 3. The low score for waste management corresponded to the absence of containers for medical non-contaminated waste (B1 containers). The deficiencies observed and consequent recommendations are summarized per category in Table 4. In the category “persons”, long hair was not systematically tied up for lab working, so it can get contaminated through direct contact or indirect contact with contaminated gloves. One person wore jewels, which may offer harbor for infectious pathogens. One person exited the lab with a lab coat on, which is not recommended as it can be contaminated. The biosafety level and the restricted access (color lines) were not specified on the lab door, as expected. Anteroom handwashing station was only disinfected once a week, instead of after each practical session. At last, no B1 waste container was present in the lab, although it is a minor deficiency if a B2 container is available.

The internal audit and observations of practices in the Virology lab highlighted much less deficiencies, as compared to the first case study.

## 4. Discussion

The present paper detailed a methodology based on checklists and a scoring system to assess the compliance with biosecurity (biosafety for labs) in different sectors of a Veterinary Faculty. Checklists developed for the performance of the biosecurity audit allowed allocating a score to each biosecurity measure classified in different categories. It is a standardized way to evaluate the compliance with biosecurity procedures, as they should normally be implemented, and to identify rapidly a category or specific measures for which compliance is not optimal. A Faculty of Veterinary Medicine is an interesting environment from a biosecurity point of view, as students play an important role and are greatly involved in the different sectors at risk from a biological point of view. It is thus important, for the benefit of all, to be sure they follow the rules. Indeed, veterinary (vet) students are a population particularly at risk in terms of infectious diseases and zoonoses, e.g., multi-drug resistant- and extended spectrum beta-lactamase-producing bacteria (12). Vet students have less experience in the application of biosecurity measures, and outbreaks of zoonotic diseases have been reported in this population in the past. In January 2007, six vet students were infected with *Cryptosporidium* spp.; the outbreak was caused by a lapse in hygiene, particularly handwashing (13). In 2013, six vet students were contaminated by *Cryptosporidium* spp. from foals hospitalized in an equine perinatology unit (14). During the same year, a more important outbreak of cryptosporidiosis involved 56 vet technology and 100 vet science students at Massey University over an 8-week period (15). Increasing awareness of vet students about zoonoses and the tools to prevent them is thus a crucial part of biosecurity teaching.

**Table 4:** Main biosecurity deficiencies observed when performing the internal audit in the virology lab, per category of items.

**Category of items**	**Score**				**Non-compliances observed**	**Risk**	**Recommandations**
**PERSONS**	**0**	**1**	**2**	**3**			
Dress and equipment	14	0	0	4	Long hair not systematically tied up	Risk of direct contact with a contaminated environment or indirect contamination (through touching hair with contaminated gloves)	Hair must be tied up
	16	0	0	2	Jewels worn	Jewels are a nest for pathogens and might create holes in the gloves.	No jewels should be worn when working in a lab
**BEHAVIOR**							
17	0	1	0	Wearing the lab coat to exit the lab, but after disposal of gloves and hand hygiene	Lab coats might be contaminated, thus risk of spreading pathogens outside the lab	Lab coats should not be worn outside of the lab
**PREMISES**							
Lab itself				1	Biosafety level not indicated on the lab door and absence of color lines (marking restricted access)	No information on the risk level and thus requirements to enter the lab	The biosafety level should be specified on the lab door, and a color line signifying restricted access should be marked in front of the lab door
		1			Anteroom handwashing station disinfected only once a week	Risk of hand contamination	Handwashing station should be disinfected after each practical work
**WASTE**							
B1 containers				1	No B1 containers (for non-biologically contaminated waste)	Would improve waste management, but no additional risk if B2 containers (for biologically contaminated waste) are present and all waste is disposed of in them.	Implement a B1 container in the lab and in the anteroom, but ensure a correct waste management.

Two staff members (one scientific and one technician) and 14 students of 2nd Bachelor in Veterinary Medicine (BVM2) students were observed. Students are split into two subgroups and each subgroup performs a BSL2 activity during one session (6 students on the first session and 8 of them during the second session, as one student was missing during the first session).

The choice of median vs. average score is also important; indeed, in both case studies, medians and averages appeared sometimes quite different. Considering advantages and disadvantages of both variables helped selecting the most appropriate in the current context, i.e., average scores. Indeed, they draw directly attention on a category to investigate and items needing improvement. In both case studies, median scores were null for several categories, while average scores were above zero: if considering median scores only, one would think that, for a given category, compliance is perfect, while an average score above zero means one or more items need improvement.

### 4.1. Class 3 of the Equine Clinic

Several wrong practices and problems linked to premises were observed and highlighted throughout the audit; they related to the following components of biosecurity (2): (i) risk of cross-contamination by wearing the same PPE (long-sleeve disposable lab coat and gloves) for two different patients–(ii) risk for human health because of inadequate hand hygiene–and (iii) leaving a contaminated effluent spread on the facility grounds. The recommendations formulated require, most of the time, minor adjustments. Awareness raising on the importance of strictly implementing barrier nursing is a critical point. Indeed, deficiencies were clearly identified for PPE and hand hygiene, two pillars of barrier nursing. Regular hand hygiene campaigns have shown to be efficient in raising awareness: as an example, a short-term public health campaign held in Kansas State University College of Veterinary Medicine and targeting veterinary students, successfully improved hand hygiene before meals: the campaign relied on short educational videos and a motivational poster (16). When caring for infectious patients, an adequate PPE provides a physical barrier to prevent the transmission of pathogens (17), especially important if they are zoonotic; furthermore, in a veterinary context, it also helps minimizing the role of a care provider in the transfer of pathogens between several patients. The standard cleaning and disinfection processes, as it should be implemented according to SOPs, needs to be reminded to the persons in charge. It is also important to remind people that eating or drinking in the clinic might represent a risk for them, especially if one or more patient is infected with a zoonotic pathogen. In this case, individuals may be infected after contaminating their hands prior to consuming food or drinks. The class of risk and infectious diagnosis should clearly be specified on the horse's box, in the horse's file, on samples and on the patient itself (wearing a colored halter for example) in order to inform on particular precautions to be taken in case of (in)direct contact with the patient, and for the decontamination of the environment. Minimizing environmental contamination when a class 3 patient is taken out of the box, i.e. cleaning and disinfection of feet before leaving the box, putting overshoes to prevent floor contamination as well as cleaning and disinfection of the horse's path if necessary, is important as well.

The efficacy of foot baths/foot mats in preventing mechanical transmission of bacteria *via* contaminated footwear is sometimes controversial, some studies having demonstrated that they were efficient, while others not (18–22). Even if the efficacy depends on the disinfectant(s) used, a common trait is that many disinfectants are inactivated by the presence of organic matter (23). Therefore, walking in a footbath with dirty shoes is useless since it adds organic matter to it, potentially inactivating the disinfectant.

Infection control implies reducing the presence of pathogens in the environment (24–26). Cleaning is a key preliminary step in the process, and should always precede disinfection (27). The cleaning and disinfection process, as standardized in the Biosecurity SOPs, should be reminded to people in charge, in order to optimize its efficient application and prevent the environmental persistence of pathogens. Disinfectants are biocides, and their handling is not harmless for users. Additional PPE such as respiratory protection and safety glasses is often required. People who use these biocides should be aware of the chemical risk, and wear the PPE recommended by the manufacturer. The contact time may vary from a disinfectant to another. The manufacturer's recommendations should be strictly followed.

Waste management is another main component of biosecurity. In both case studies, B1 containers were not present in facilities. Only B2 waste containers were present, it is to say even non-contaminated medical waste had to be disposed of in B2 containers. That is not a problem from a biosecurity point of view, as it corresponds to the implementation of an over-adapted procedure. Indeed, B2 containers are all incinerated. By contrast, sharp management was not appropriate: sharp handling is important to prevent needle stick injuries (28). Unsafe needle-handling practices must be reduced and training programs could encourage safe needle-related practices (29).

Equipment may act as a mechanical vector. A correct cleaning and disinfection process is thus important as well, in order to prevent such risk. For example, stethoscopes are routinely in contact with patients and are potential vectors for hospital-acquired infections (30–32).

### 4.2. Virology lab practical activities

The co-existence of two activities of a different risk category in a same lab, i.e., BSL2 (using a biosafety cabinet) and BSL1 (microscope observation, with note taking) could be a source of biosecurity breaches and crossed contaminations, thus requiring more attention from the supervisors. Nevertheless, the group size allows the BSL2 activity-supervisors to guide the students strictly. Rules implemented in the lab were globally well complied with by students, partly thanks to the strict supervision and guidance, step-by-step, by supervisors. The worst median score obtained for waste management did not have any consequences in terms of biological safety, as all waste were disposed of in B2 containers (dedicated to biologically contaminated waste) that were destroyed after collection.

If the two case studies are compared, it appears that compliance in the Equine Clinic class 3 was not as good as for the practical virology labs. A difference in supervision could greatly explain such difference. Indeed, while students were strictly supervised and guided step-by-step by the staff in the virology lab, they were often left under the supervision of young clinicians (interns) in the Equine class 3. These clinicians may have been more concerned about the patients' health than the strict application of all biosecurity measures. Apart from raising awareness among young clinicians, making the students responsible for the application of biosecurity measures in the clinic could also improve compliance. One way to motivate students to follow biosecurity measures could be to evaluate them on the basis of their biosecurity behavior, i.e. they would be rated (by receiving bonus points for example) according to their diligence in following biosecurity rules while working in the clinics. Several recommendations were provided, and it will be interesting to repeat the audit in that sector in the future to assess the hopefully positive evolution of scores.

The methodology described in the present paper allows assessing the implementation of biosecurity SOPs in a specific sector and/or for a given activity. The identification of criteria needing improvement is a key point: it helps prioritizing actions to be implemented and awareness raising among all people concerned. Regular internal auditing is an essential part of a biosecurity plan. Standard operating procedures should be promoted and need regular audits to monitor its practical application and to identify any shortcomings that could compromise the level of biosecurity. When a shortcoming is identified, corrective action should be taken (15). For example, biosecurity audits were proven to be crucial in the control of laryngotracheitis in broilers (33). Audit and annual monitoring also increases general awareness, as stated recently by Vercken and Paillot (34) in the context of equine infectious diseases.

The repetition of internal audits over time can only promote continuous improvement of the program, as it allows a follow-up of the situation and the checking of critical points and deficiencies highlighted in a previous audit. The follow-up of observations through time is useful to assess the evolution of compliance with biosecurity measures (35). The frequency of audits is risk-dependent. A high-risk area or activity, i.e. where infectious hazards are well assessed (e.g., the isolation unit), should be audited on a more frequent basis (e.g., perhaps quarterly or twice a year). On the other hand, low-risk areas or activities could be audited once a year. Besides, the time dedicated to observations depends on the area or activity itself. The compliance rate is another criterion to help determining the frequency of audits (36): if not satisfactory, and improvement is needed, the audit could be performed more often in order to assess the implementation of requirements. The time dedicated to observations will depend on. In both examples developed in the present paper, the duration of observations was conditioned to the length of time students were present in the equine clinic (3.5 h) or performing the practical virology sessions.

The present methodology allows assessing the compliance with biosecurity rules, as they should be implemented, according to the Biosecurity SOPs. The application of a scoring system highlighted that several components of biosecurity were not complied with. Critical points where action is needed were identified and recommendations were provided and further discussed with the responsible staff in both sectors, in order to build an improvement action plan. Corrective actions should be prioritized before implementation and awareness should be raised among all people concerned. The repetition of audits, and the follow up of scores over time, will allow figuring out whether recommended changes help improving compliance scores. The methodology presented here is original and could be applicable to other contexts, e.g., in the context of evaluation of Veterinary Education Establishments by the European System of Evaluation of Veterinary Education (ESEVT). In addition, involving students in the process of audits in veterinary school clinics will contribute to their education and to audit training, for example in farms, when they enter their professional life.

## Data availability statement

The original contributions presented in the study are included in the article/Supplementary material, further inquiries can be directed to the corresponding author.

## Author contributions

M-FH: conceptualization, methodology, performance of audits, and writing—original draft preparation. CS: supervision, validation, and writing—review. Both authors contributed to the article and approved the submitted version.
